# Prediction of Labor Unemployment Based on Time Series Model and Neural Network Model

**DOI:** 10.1155/2022/7019078

**Published:** 2022-06-03

**Authors:** Xitao Liu, Lihui Li

**Affiliations:** School of Public Finance and Administration, Harbin University of Commerce, Harbin 150001, Heilongjiang, China

## Abstract

With the advent of big data, statistical accounting based on artificial intelligence can realistically reflect the dynamics of labor force and market segmentation. Therefore, based on the combination of machine learning algorithm and traditional statistical data under big data, a prediction model of unemployment in labor force based on the combination of time series model and neural network model is built. According to the theoretical parameters, the algorithm of the two-weight neural network is proposed, and the unemployment rate in labor force is predicted according to the weight combination of the two. The outcomes show that the fitting effect based on the combined model is superior to that of the single model and the traditional BP neural network model; at the same time, the prediction results with total unemployment and unemployment rate as evaluation indexes are excellent. The model can offer new ideas for assisting to solve the unemployment of the labor force in China.

## 1. Introduction

Unemployment is not only a comprehensive economic problem but also a complex social problem [1] which has turned into the focal point of consideration for all nations. Whether unemployment can be properly solved is related to the development of the country. In recent years, China's economy is facing a complicated international economic environment, such as RMB appreciation [2], prices rising of raw materials [3], and pressure increase of inflation input [4], which has led to the decline of export and economic growth, thus bringing about unemployment that deserves our attention. In addition, the government also attaches great importance to the development of a country, which depends not only on the country's economy, science, technology, military affairs, and the gap between the rich and the poor but also on the treatment results of unemployment. If countries with serious unemployment do not have effective policies and measures to ensure the survival of the unemployed labor force, it can easily lead to social instability, which will affect the development of a country in all aspects. Therefore, if the government can predict the number and proportion of unemployed labor force in advance, they can formulate policies and measures to ensure the prosperity and development of the country according to these predicted data [5].

At present, with the advent of big data, some researchers try to use circumstantial big data to build indicators for measuring the dynamics of China's labor market [6–8]. For example, by using recruitment data on Internet [9], social media data [10], data of digital search [11], etc., to build economic indicators based on nonstatistical accounting methods, which can reflect the dynamics of the whole labor force and market segments in real time. However, these indicators lack a clear definition in the accounting sense, and their scientific and reliability are questioned. For the method of prediction, because the urban unemployment and unemployment rate in China are all differential stationary time series data, the neural network algorithm has been proved to have an excellent approximation ability to nonlinear relationship in theory [12, 13], so it is of practical significance to study a prediction model of unemployment in the labor force, so as to solve the actual unemployment.

Before 2018, the most important unemployment index released by the Chinese government was the registered urban unemployment rate, which was quite different from the international definition of the unemployment rate, and the value remained stable at around 4% for a long time, so it was difficult to reflect the real unemployment level in China [14]. Since 2018, the Chinese National Bureau of Statistics has released the national urban survey of the unemployment rate on a monthly basis, which is a significant progress in the release of unemployment statistics. To make up for the long-term lack of China's four macro-economic indicators, the data have been generally recognized by all sectors of society. However, due to the limited sample size of the labor force survey, the unemployment rate in the urban survey is not representative of administrative regions at all levels below the provincial level [13]. In 2021, the Chinese National Labor Force Survey will conduct a new round of sampling according to the seventh national census and appropriately expand the sample size to meet the main index data, such as the urban unemployment rate, which is well representative of the country and provinces (autonomous regions and municipalities).

Therefore, in this paper, the above-mentioned combination model is established, and the unemployment situation of the urban labor force with the number of unemployed and unemployment rate as evaluation indexes to explore new ideas that are helpful to manage the urban unemployment in China.

## 2. Related Theories

### 2.1. Neural Network Model

#### 2.1.1. BP Neural Network

**Figure 1:**
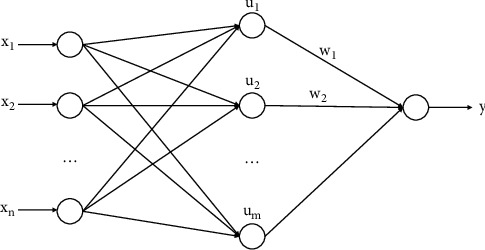
Topology of the BP neural network.

First proposed by the Parallel Distributed Processing scientific research group represented by Rumelhart and Mccelland in the United States, BP neural network was is a multilayer perceptron feedforward network [15]. It has strong nonlinear adaptability, excellent nonlinear approximation, and good fault tolerance. Therefore, it can process large-scale data in parallel with outstanding self-organizing, self-learning, and self-adapting abilities, which are widely adopted in the fields of prediction, classification, and clustering. This back propagation algorithm makes multilayer perceptron has the ability to approximate any complex nonlinear function [16]. It is a feedforward neural network using the BP learning algorithm, and Figure 1 shows its topology.

It can be seen that the direction of data flow is that first the data of the input layer flow to the first hidden layer after weighting, then the data flow to the second hidden layer after further weighting, and so on until the output data of the last hidden layer flow to the output layer. The change function between the info and the result of neurons in each layer is known as the actuation work. The universal approximation theorem has been proved [17, 18]. A three-layer BP neural organization with a secret layer can surmise any ceaseless capacity in a limited locale with erratic accuracy as long as there are an adequate number of stowed away hubs. The function corresponding to the *i*th neuron in a certain layer is shown in the following formula:

(1) $$\begin{array}{c} y_{j}=f\left(W_{J}*X-\theta_{j}\right)\\ =f\left(\overset{n}{\underset{i=1}{\sum }}\omega_{ij}x_{i}-\theta_{j}\right). \end{array}$$

In which, *W*_*J*_ is the weight in the BP neural network, *X* is the input vector, *θ*_*j*_ is the threshold, and *F* is the activation function.

#### 2.1.2. RBF Neural Network

**Figure 2:**
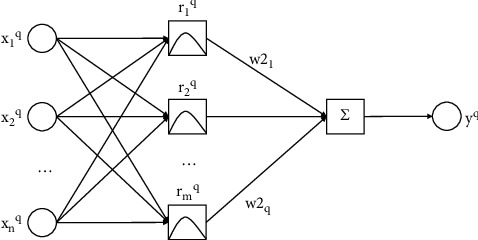
Topology of RBF neural network.

The radial basis function neural network (RBF) also belongs to feedforward neural network. It is widely used in function approximation and classification its straightforward design, simple preparation, quick learning combination, and the capacity to estimate any nonlinear capacity [19]. Although RBF has only one hidden layer, it has the same excellent nonlinear approximation ability as BP neural network, and Figure 2 shows its topology.

The basis function of hidden nodes in RBF adopts the distance function, and the activation function adopts the Gaussian function. Since RBF has spiral evenness about a middle mark of N-layered space, and the farther the contribution of neurons is from the middle point, the less activated the neurons are. This characteristic is often called “local property” [20].

#### 2.1.3. Two-Weight Neural Network

For the two-weight neural network, by changing parameters of neuron function, it can also be constructed not only as a higher-order neural network [21]. In addition, the neural of the double-weight neural network includes not only the weights of the BP neural network but also the core values (i.e., central data). The neuron function of the neural network is shown in the following formula:

(2) $$\begin{array}{c} y_{j}=f\left(\sum_{i=1}^{n}{\left(\frac{\omega_{ij}\left(x_{i}-c_{j}\right)}{\left|\omega_{ij}\left(x_{i}-c_{j}\right)\right|}\right)}^{s}{\left|\omega_{ij}\left(x_{i}-c_{j}\right)\right|}^{p}-\theta \right). \end{array}$$

Among them, *y*_*i*_ is the output value of the *j*th neuron in a layer in the two-weight neural network model, *f* is the activation function, *ω*_*ij*_ is the weight of neuron, *c* is called the kernel value of neuron, *x*_*i*_ is the input value of neuron, *θ* is the threshold value of neuron, and *s* and *p* are two parameters.

In formula (2), when *C*_*j*_ = 0, *S* = 1, and *p* = 1, it becomes the neuron function of the BP neural network; When *s* = 0 and *p* = 2, the function becomes the neuron function of RBF neural network. For the hidden layer, the neuron function of this layer only contains the direction weight but not the core weight. For the hidden layer of the RBF neural network, the neuron function of this layer only contains core weights but does not contain the direction weight; while for the hidden layer of the two-weight neural network, the neural function of this layer contains both the direction weight and the core weight. Therefore, when the parameters of the activation function take specific values, it has more excellent approximation ability for nonlinear continuous functions [22].

In view of this, the algorithm of the two-weight neural network is taken. To apply all training samples to the improved BP algorithm of weight adjustment (batch processing) in the neural network. The minimum mean square error criterion is selected to evaluate the learning rule and its objective function is defined as the following equation:

(3) $$\begin{array}{c} E^{\left(p\right)}=\frac{1}{2}\sum_{l=0}^{m-1}{\left(d_{l}^{\left(p\right)}-y_{l}^{\left(p\right)}\right)}^{2}. \end{array}$$

The total error of all input samples is

(4) $$\begin{array}{c} E_{T}=\sum_{p=1}^{P}E^{\left(p\right)}\\ =\frac{1}{2}\sum_{p=1}^{P}\sum_{l=0}^{m-1}{\left(d_{l}^{\left(p\right)}-y_{l}^{\left(p\right)}\right)}^{2}, \end{array}$$

where *P* is the number of learning sample vectors, *d*_*l*_^(*p*)^ (*p* = 1,…,*P*) is the expected output, and*y*_*l*_^(*p*)^ (*p* = 1,…,*P*) is the actual output.

Generally, the gradient descent method is used to modify the weights. In this paper, the formulas for adjusting the weights and the core values are as follows:

(5) $$\begin{array}{c} \omega \left(t+1\right)=\omega \left(t\right)-\left(1-\alpha \right)\eta \frac{\partial E_{T}}{\partial \omega \left(t\right)}+\alpha \Delta \omega \left(t\right),\\ c\left(t+1\right)=c\left(t\right)-\left(1-\alpha \right)\eta \frac{\partial E_{T}}{\partial c\left(t\right)}+\alpha \Delta c\left(t\right). \end{array}$$

Among them, *η* represents the learning rate whose value is generally small, Δ*w*(*t*)=*w*(*t*) − *w*(*t* − 1), Δ*c*(*t*) = *c*(*t*) − *c*(*t* − 1), *α* is the momentum factor, and the value of *α* is

(6) $$\begin{array}{c} \alpha =\left\{\begin{array}{cc} 0, & \text{SS}\left(t\right)>1.04\text{RSS}\left(t-1\right),\\ 0.95, & \text{RSS}\left(t\right)<\text{RSS}\left(t-1\right),\\ \alpha , & \text{others}, \end{array}\right) \end{array}$$

where RSS() is the residual sum of squares of the output.

The learning of network is divided into two processes [23, 24]: one is to calculate the output of each layer by forward propagation from front to back and the other process is to correct the weight or core value of each layer by backward propagation from back to front. The Algorithm 1 steps are as follows:

### 2.2. Time Series Model

The time series of a random event usually refers to sequence {*X*_*t*_}, formed by arranging the numerical values of an index observed at equal intervals in a certain period of time in sequence, which essentially reflects the trend that an index changes with time. The main purpose of time series analysis is to observe and study the existing data, mine the development law of these data with time, and thus analyze and estimate its future situation in the time series model [25]. The analyzed data are differential stationary time series in nonstationary time series. The so-called time series is a series of ordered data recorded according to the time sequence. In daily life, the data of time series can be seen everywhere, and most of these data are nonstationary time series, among which the ARIMA model, as a kind of nonstationary time series analysis, is widely used.

ARIMA (*p*, *d*, *q*) model has the following formula:

(7) $$\begin{array}{c} \left\{\begin{array}{c} \left(1-\Phi_{1}B-\cdots -\Phi_{p}B^{p}\right)\nabla^{d}x_{t}=\left(1+\theta_{1}B+\cdots +\theta_{q}B^{q}\right)\varepsilon_{t},\\ E\left(\varepsilon_{t}\right)=0,\mathrm{var}\left(\varepsilon_{t}\right)=\sigma_{\varepsilon }^{2},\\ \begin{array}{c} \mathrm{cov}\left(\varepsilon_{t},\varepsilon_{s}\right)=0\left(s\neq t\right),\\ \mathrm{cov}\left(X_{s},\varepsilon_{t}\right)=0\left(\forall s<t\right). \end{array} \end{array}\right) \end{array}$$

The modeling steps can be summarized as [26, 27]follows:

1. Test the stationarity of the original sequence data. There are two most commonly used methods, one is the graph test, and other is the unit root test. If the sequence is stationary, the graph of data changing with time should show that most of the data fluctuate at a constant and its fluctuation range is similar, autocorrelation coefficient will quickly decay to zero. When the sequence is nonstationary, the corresponding characteristic equation contains characteristic roots. Therefore, the unit root test can also be used to verify whether the sequence is stationary. The commonly used ones are DF, ADF test, etc. If the sequence is not stationary, the difference operation will be carried out.
2. Test the white noise of the series. It is not meaningful for all stationary series models, but only for those series models that can influence each other.
3. Determine the model's order. The model to be established and its order can be determined according to the correlation coefficient diagram of stationary time series, and the order can also be determined according to the identification function.
4. Parameter estimation: The AIC criterion can be used to complete the process of order determination and parameter estimation simultaneously.
5. Model test. There are usually two types of tests: the significance test of model, whose purpose is to test whether the established model effectively and adequately extracts the information contained in the data. If the established model is effective, the residual has the characteristics of white noise data, and there is no available information; Otherwise, it means that the information in the data has not been extracted completely, and the significance test of the parameters remaining in the residual error is to confirm whether each parameter obtained is obviously different from zero to establish a simpler and more accurate model.

## 3. The Combined Model of Unemployment Prediction in Labor

Based on the above sample data, we first forecast the number of registered unemployed people and the registered unemployment rate in China by using the two-weight neural network and then forecast the data by using the time series method. Finally, the predicted results of the two methods are combined according to the corresponding weights as the predicted values of the combined model.

### 3.1. Two-Weight Neural Network Model

In this paper, the parameters in the neurons of the double-weight neural network are *s* = 0, *p* = 1, *θ* = 0, and the BP algorithm is used. When taking two-weight neural network to analyze, it is important to standardize the information first. The normalization formula for the series of the number of unemployed and unemployment rate is:

(8) $$\begin{array}{c} x\left(i\right)=2\frac{x_{0}\left(i\right)-x_{0\min}}{x_{0\max}-x_{0\min}}-1. \end{array}$$

Among which, *X*(*i*) is the *i*th value in the normalized sequence, *x*_0_(*i*) is the original data, *x*_0min_ is the minimum value, and *x*_0max_ is the maximum value.

A training sample is constructed with the normalized data of unemployed and unemployment rate, and the method of sample construction is shown in the following formula:

(9) $$\begin{array}{c} X_{i}=\left(x\left(i\right),x\left(i+1\right),x\left(i+2\right)\right),\\ Y_{i}=x\left(i+3\right). \end{array}$$

In which, *X*_*i*_ and *Y*_*i*_ are the samples *i*th group, and *x*(*i*) is the *i*th value of the normalized data. The sample data of nearly 30 years are selected in this model, and Table 1 shows some normalized samples as follows:

Through the prediction method of two-weights neural network, the last expectation results are displayed in Table 2.

### 3.2. Time Series Model

Aiming at analyzing the data that changes with time, the time series analysis uses the time series model to analyze the unemployment and the registered unemployment rate in cities and towns. Because the data series of labor unemployment is nonstationary, it is necessary to make a first-order difference to the original series, and the series after the difference has a very strong short-term correlation. Therefore, the ARIMA model is selected for fitting.

The statistical samples are tested for significance level of *a* = 0.05. The white noise test of the first-order differential sequence of unemployed shows that the *P* value of the statistics constructed after the sixth-order delay is 0.0122, which is less than 0.05, while for the unemployment rate, the *P* value is 0.0017 that less than 0.05. It can be considered that the differential sequence is a nonwhite noise sequence; that is, the related information of the differential sequence cannot be ignored and has yet to be extracted. The ARIMA model can be used to fit the differential stationary and nonwhite noise sequence.

After the difference sequence, the autocorrelation coefficient is trailing, so as the partial autocorrelation coefficient. After testing, when the ARIMA model with *p* = 1 and *q* = 0 is adapted to fit the data, the best results are obtained, and the upsides of AIC and SBC compared to the model are additionally the smallest. The results are shown in Table 3.

Under the significance test level of 0.05, the residual sequence test results are shown in Table 4.

The *P* values of the statistics constructed are far greater than 0.05, so it can be considered that the residual sequence no longer has the significance of extracting information. The results of the coefficient test and residual test show that the ARIMA (1, 1, 0) model implemented in this paper has a decent displaying impact on the sequence of unemployment.

According to the established model, the unemployment of the labor force is predicted, and the prediction results are shown in Table 5.

### 3.3. The Combined Model

**Figure 3:**
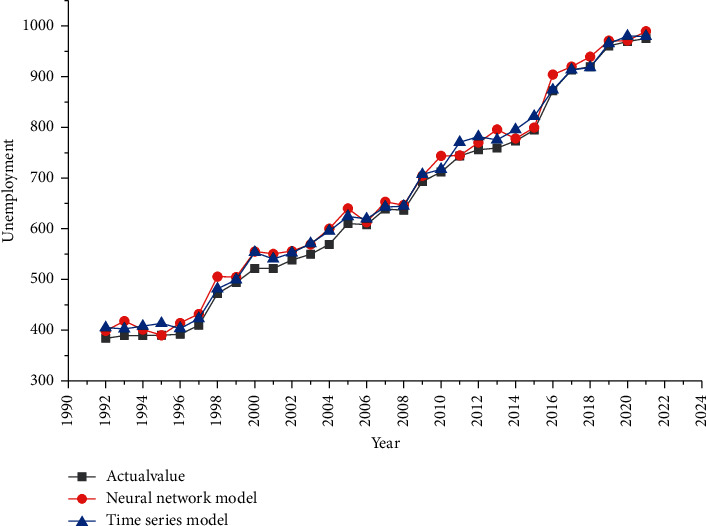
Fitting chart of unemployment under single model.

**Figure 4:**
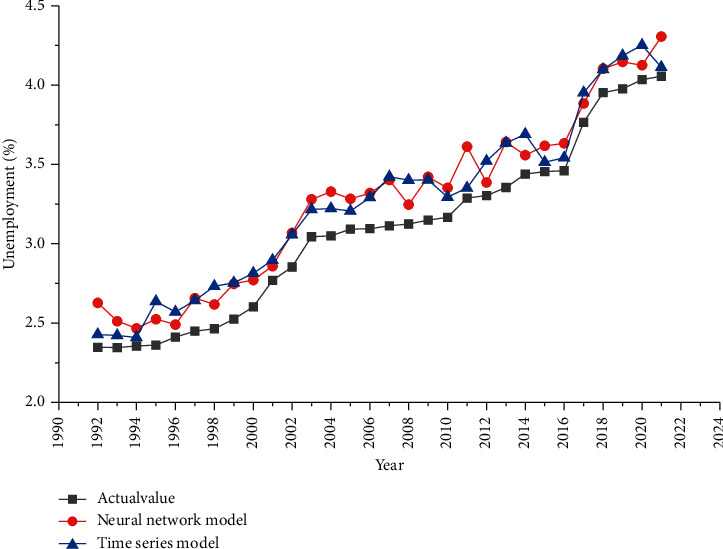
Fitting chart of unemployment rate under single model.

In the previous part, the two-weight neural network model and the ARIMA model were used to analyze the number of unemployed and the unemployment rate in China. The two analysis methods are suitable for fitting the series of unemployed laborers and unemployment rate in China. The fitting values of the two models about the sample data are shown in Table 6:

Figures 3 and 4 describe the fitting situation between the number of unemployed and the unemployment rate under a single model. From the figure, it can be inferred that the fitting value of neural network model and time series model is higher than the actual value, but the curves of the three models are similar, showing that the fitting of single model is better. But for the unemployment rate, there is a certain deviation between the fitting curve and the actual curve.

According to the fitting results of the unemployment model, the sum of residual squares between the fitting value and the true value is solved, which is shown in Table 7:

Assuming that *y*_0_ represents the true value, *y*_1_ represents the fitting value of the neural network model, *y*_2_ represents the fitting value of the time series model, *W* represents the residual value corresponding to the combination model, *ω* (0 ≤ *ω* ≤ 1) represents the weight of the neural network model in the combined model, and 1-*ω* represents the weight of the time series model in the combined model. The combined model is calculated by the following formula:

(10) $$\begin{array}{c} \left\{\begin{array}{c} W_{\min}={\sum_{k}^{i=1}\left(\omega y_{1}+\left(1-\omega \right)y_{2}-y_{0}\right)}^{2},\\ 0\leq \omega \leq 1, \end{array}\right) \end{array}$$

where *k* is the number of true values for calculating the residual sum of squares.

After calculation, when *ω* is 0.4267, the minimum residual error is 21353. Similarly, when *ω* is 0.598, the minimum residual error of the combined model is 0.6574, which is smaller than the residual sum of squares fitted by neural network and time series model. According to this weight, the predicted value of the combined model for unemployment is shown in Table 8:

### 3.4. Other Comparison

Because the fitting effect of the combined model is better than that of the single model, which constitutes the combined model, it is still necessary to compare whether the fitting effect is better for other models. The BP neural network model, the most commonly used network model, is selected as the prediction model. For the number of unemployed, the minimum residual error of BP neural network model fitting is 25,673, and for the unemployment rate of labor force, the minimum residual error is 2.9760. Both of them are higher than the minimum residual error obtained by the combined model in fitting, which shows that in the model built in this paper, there are better fitting results and it seems to be more accurate to predict the unemployment in labor force.

## 4. Conclusion

An accurate prediction of unemployment in labor force is helpful to tackle the issue of metropolitan unemployment. In this paper, the data of unemployment obtained by the Chinese National Bureau of Statistics are taken as a statistical sample. A labor unemployment prediction model in light of the combination of the neural network model and time series model is built, while the situation about labor unemployment in China from 2022 to 2030, is forecasted. The results show that the fitting impact of the combined model is superior to that of the single model, which constitutes the combined model. The minimum residuals of the combined model for fitting and the unemployment rate are 21353 and 0.6574, respectively, which are lower than their single models. At the same time, the fitting condition of the combined model is better than that of the commonly used BP neural network model, which has an excellent impact. When the latter is fitted to the unemployed and unemployment rate, the obtained minimum residuals are 25673 and 2.9760, respectively, which are higher than the combined model. The prediction results of the combined model show that from 2022 to 2030, the number of unemployed labor force in China will fluctuate between 999.6992 and 1038.8520, and the unemployment rate will vary between 5.3406 and 5.8499.

## Figures and Tables

**Algorithm 1:**
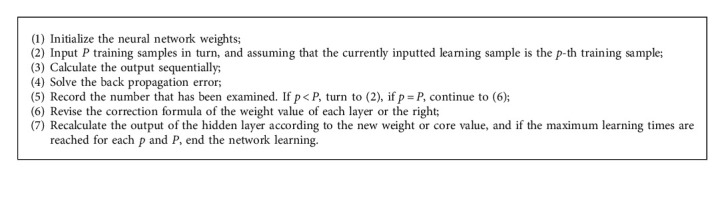
Process of learning of network.

**Table 1:** Normalized data of sample.

	Unemployment	Unemployment rate
1992	−0.65643843	−0.722222222
1993	−0.505828755	−0.444444444
1994	−0.354343843	−0.388888889
1995	−0.239179954	−0.277777778
…	…	…
2018	0.801688329	0.277777778
2019	0.839206753	0.277777778
2020	0.825807316	0.277777778
2021	0.919603377	0.277777778

**Table 2:** Prediction results of unemployment based on two-weight neural network.

	Unemployment (ten thousand people)	Unemployment rate (%)
2022	1076.6072	5.8051
2023	1104.8578	5.7644
2024	1074.7537	5.7468
2025	1114.4804	5.7465
2026	1114.0756	5.8420
2027	1081.8054	5.8074
2028	1082.2029	5.7975
2029	1073.9059	5.7892
2030	1093.9167	5.8136

**Table 3:** Fitting results of time series model.

	Unemployment	Unemployment rate
Parameter	AR(1)	AR(1)
Coefficient	0.59849	0.59901
Standard error	0.10207	0.10189
Statistic	4.53	4.54
*P* value	<0.0001	<0.0001
AIC	399.7305	8.926
SBC	481.4685	9.564

**Table 4:** Residual sequence test.

Latency	*Q*_LB_ statistic		*P* value	
	Unemployment	Unemployment rate	Unemployment	Unemployment rate
6	1.4	1.93	0.9260	0.8582
12	7.9	6.15	0.8652	0.7982
18	10.2	8.65	0.7289	0.8321
24	19.2	15.06	0.8729	0.7765

**Table 5:** Forecast results of unemployment based on time series model.

	Unemployment (ten thousand people)	Unemployment rate (%)
2022	1023.087	5.5954
2023	965.6409	5.3656
2024	982.5995	5.6965
2025	970.6785	5.5118
2026	1004.326	5.3388
2027	956.5798	5.6677
2028	1035.294	5.7514
2029	1030.214	5.3738
2030	1023.087	5.5954

**Table 6:** Fitting results of unemployment in labor force under different models.

	Unemployment			Unemployment rate		
	Actual value	Neural network model	Time series model	Actual value	Neural network model	Time series model
1992	363.9	426.06	353.64	2.3	2.59	2.18
1993	420.1	433.63	390.90	2.6	2.51	2.30
1994	476.4	470.81	473.73	2.8	2.55	2.77
1995	519.6	521.65	530.09	2.9	2.68	2.91
…	…	…	…	…		…
2018	926	953.03	934.00	4.1	4.13	4.3
2019	952	958.69	951.38	4.1	4.13	4.3
2020	966	985.38	987.56	4.1	4.13	4.2
2021	982	998.52	994.37	4.1	4.13	4.2

**Table 7:** Residual sum of squares of fitting results.

	Neural network model	Time series model
Unemployment	24127	26370
Unemployment rate	0.9736	1.2987

**Table 8:** Prediction of unemployment based on combination model.

	Unemployment (ten thousand people)	Unemployment rate (%)
2022	1039.7289	5.5954
2023	999.6992	5.4756
2024	1010.085	5.7538
2025	1011.403	5.6462
2026	1037.8668	5.8499
2027	1023.6391	5.6047
2028	1038.852	5.3406
2029	987.4474	5.3701
2030	1012.404	5.7903

## Data Availability

The dataset can be accessed upon request.
